# Standing Up to Deconditioning: A Multidisciplinary Two-Cycle Audit to Enhance Patient Mobilization in Elderly Inpatients

**DOI:** 10.7759/cureus.110099

**Published:** 2026-06-02

**Authors:** Moe Moe San, Shwe San, Sidak Hanspal, Hind Abdelrahman, Tom Bartlett

**Affiliations:** 1 Geriatrics, Poole Hospital, Poole, GBR; 2 Gastroenterology, Poole Hospital, Poole, GBR; 3 Endocrinology, University Hospitals Dorset NHS Foundation Trust, Poole, GBR

**Keywords:** ageing and frailty, deconditioning, early mobilization, elderly inpatient, multidisciplinary teams

## Abstract

Background

Deconditioning is a common and preventable cause of functional decline among hospitalized elderly patients, contributing to prolonged recovery, increased dependence, and hospital-associated complications. Despite this, early mobilization is often limited by ward routines, invasive devices, and competing clinical priorities.

Objectives

This two-cycle audit aimed to evaluate whether low-cost, multidisciplinary interventions could improve early mobilization among elderly inpatients and reduce the risk of deconditioning.

Methods

A baseline audit was conducted on an elderly inpatient ward to assess the proportion of eligible patients mobilizing out of bed by 11:00 a.m. daily. Findings informed two sequential Plan-Do-Study-Act (PDSA) cycles. Cycle 1 focused on promoting early mobilization during morning handovers and minimizing unnecessary barriers such as intravenous lines and urinary catheters. Cycle 2 introduced sit-out charts and educational posters to improve documentation and sustain engagement. Data were collected daily at a fixed time point, excluding patients receiving end-of-life care or those who were permanently bedbound.

Results

At baseline, a mean of 7.73 patients (30%) were mobilizing out of bed daily. Following PDSA Cycle 1, this increased to a mean of 14.71 patients (57%). During Cycle 2, improvement was sustained, with a mean of 13.14 patients (51%) mobilizing daily. Staff feedback indicated improved awareness and shared responsibility for patient mobility.

Conclusion

This two-cycle audit demonstrates that simple, low-cost, multidisciplinary interventions embedded into routine ward practice can sustainably improve early mobilization among elderly inpatients. Early mobilization should be considered a core marker of quality inpatient care and a key strategy in reducing the risk of hospital-associated deconditioning.

## Introduction

Hospital-associated deconditioning (HAD) refers to the decline in physical and/or cognitive function that occurs in older adults during or after hospitalization and is a well-recognized, potentially preventable cause of functional deterioration [1]. It is estimated that 30-60% of hospitalized older adults experience some degree of functional decline during their hospital stay, regardless of the severity of the admitting illness [1-3]. Reduced mobility, prolonged bed rest, and restrictive hospital environments can lead to rapid loss of muscle strength and physical capacity within days of admission [4].

The effects of immobility on older adults are well documented. Experimental studies show that 10 days of bed rest can result in approximately 10% loss of lean body mass, accompanied by reductions in lower-extremity strength and metabolic reserve [5]. Observational studies further demonstrate that hospitalized older adults spend the majority of their time inactive, with some studies reporting that over 80% of their time in a hospital is spent in bed, even among patients who were independently mobile prior to admission [6,7]. Low in-hospital mobility has therefore been identified as a key and modifiable contributor to hospital-associated functional decline [6].

The clinical consequences of HAD are substantial. Approximately one-third of older hospitalized patients experience a decline in activities of daily living during admission, and many fail to regain their baseline function before discharge [2]. Functional decline during hospitalization is associated with longer hospital stays, increased risk of institutionalization, higher fall risk, and increased mortality following discharge [2,3,7]. Lower levels of physical activity during hospitalization are also linked to poorer discharge outcomes and reduced likelihood of returning home independently [8].

Recognizing these risks, professional organizations emphasize the importance of early mobilization for hospitalized older adults. The British Geriatrics Society promotes the principle that preventing deconditioning is “everyone’s responsibility,” encouraging healthcare professionals to support patients in sitting out of bed, wearing day clothes, and maintaining activity during hospitalization [9-11]. Similarly, the American Academy of Nursing Choosing Wisely initiative advises clinicians not to allow older adults to remain confined to bed during their hospital stay [12].

Evidence supports the safety and effectiveness of mobilization-focused interventions in hospital settings. Multidisciplinary and nurse-led mobility programs have been shown to increase out-of-bed activity, improve walking ability at discharge, and reduce hospital length of stay without increasing fall risk [13]. Systematic reviews further indicate that hospital-based exercise and mobilization interventions improve functional mobility and physical performance without increasing adverse events, highlighting early mobilization as a cost-effective strategy for older inpatients [14].

Despite this evidence, early mobilization is often difficult to implement consistently in routine inpatient care. Acute care environments frequently prioritize medical stabilization over functional preservation, while hospital routines, including prolonged use of bed gowns, urinary catheters, intravenous lines, and continuous monitoring, may unintentionally promote inactivity [15,16]. In addition, mobility is sometimes perceived as the responsibility of physiotherapists alone, resulting in missed opportunities for nurses and physicians to encourage daily activity [9-11].

Quality improvement (QI) initiatives may therefore play an important role in addressing hospital-associated deconditioning by embedding mobility into routine clinical practice. The present study describes a two-cycle clinical audit using the Plan-Do-Study-Act (PDSA) framework aimed at improving early mobilization among elderly inpatients on an acute geriatric ward. Following a baseline audit of mobilization practices, targeted multidisciplinary interventions were implemented to promote early mobilization and reduce modifiable barriers to activity. This study evaluates the impact of these interventions and explores their role in fostering a sustainable culture of mobility in inpatient geriatric care.

## Materials and methods

Study design and setting

This study was conducted as a two-cycle clinical audit using QI methodology based on the PDSA framework. The project was implemented on an elderly inpatient ward within a National Health Service (NHS) hospital. The ward primarily manages older adults admitted with acute medical conditions, many of whom are at increased risk of hospital-associated deconditioning due to frailty, multimorbidity, and reduced baseline mobility.

The project began with a baseline audit to assess existing patient mobilization practices. Findings from this audit informed the implementation of two sequential PDSA cycles aimed at improving early mobilization through low-cost, multidisciplinary interventions embedded into routine ward practice.

Study population

The study population included elderly inpatients admitted to the 26-bed ward during the audit and intervention periods. The ward population typically consists of patients aged 75 years and older, with a high prevalence of frailty and a representative mix of male and female patients. The project was conducted over a total of 30 days. This included a baseline audit period of 11 days, followed by PDSA cycle 1 (7 days) and PDSA cycle 2 (7 days). The transition periods between the cycles were utilized for data review, multidisciplinary feedback, and implementation of the subsequent interventions. 

Patients were considered eligible for mobilization assessment if they were: (i) receiving active medical treatment, (ii) medically optimized while awaiting discharge, or (iii) clinically stable but under ongoing inpatient care.

Patients were excluded if they were: (i) receiving end-of-life or palliative care, or (ii) permanently bedbound due to advanced neurological or musculoskeletal conditions where mobilization was not clinically appropriate.

These criteria ensured that only patients for whom mobilization was feasible and clinically appropriate were included in the analysis.

Eligibility for mobilization assessment was reviewed daily during the audit and intervention periods, as clinical suitability for mobilization could change over the course of admission.

Data collection and outcome measures

Data on patient mobilization were collected daily at 11:00 a.m., a time selected for consistency within routine ward workflows. On our ward, 'early mobilization' refers to do both: getting patients moving as soon as possible after admission and ensuring they are out of bed early in the daily routine. We chose the 11:00 a.m. time point specifically because it is when our staff aims to have patients sitting out and ready for lunch. Checking at this time allowed us to measure if this is 'early' culture was actually happening for every patient on the ward. This timing allowed assessment after morning handover and early nursing activities while minimizing interference with ward rounds and therapy sessions. In this study, “early mobilization” was defined as mobilization out of bed by 11:00 a.m., representing mobilization during the early daytime period following morning handover and routine ward care.

The primary outcome measure was the number and proportion of eligible patients who had mobilized out of bed at any time prior to or by 11:00 a.m. Mobilization was defined as either sitting out of bed in a chair or ambulating with or without assistance.

Mobilization status was assessed through direct observation on the ward and confirmed with nursing staff when necessary.

Baseline audit data were collected prior to implementation of any intervention. Following each PDSA cycle, mobilization data were collected using the same method to allow comparison with baseline performance.

In addition to quantitative data, informal qualitative feedback was obtained from members of the multidisciplinary team, including nurses, physiotherapists, occupational therapists, and healthcare assistants. Feedback focused on barriers to mobilization, feasibility of the interventions, staff engagement, and sustainability of practice changes.

Quality improvement framework

The project followed a structured PDSA framework. Each cycle was designed based on baseline audit findings and feedback from frontline staff, with the aim of addressing modifiable barriers to early patient mobilization (Table 1).

**Table 1:** Interventions Implemented During the PDSA Cycles PDSA: Plan-Do-Study-Act

PDSA Cycle	Intervention	Description
Cycle 1	Mobility-focused morning handover	Daily handover discussions included explicit mobility goals and identification of patients suitable for early mobilization.
Cycle 1	Reduction of unnecessary clinical tethers	Regular review and removal of intravenous fluids, intravenous antibiotics, and urinary catheters when clinically appropriate to facilitate mobility.
Cycle 2	Sit-out chart	A bedside chart was introduced to document daily mobilization and identify patients who had not sat out of bed for consecutive days.
Cycle 2	Educational posters	Posters placed throughout the ward highlighted the risks of deconditioning and encouraged early mobilization among staff, patients, and families.

PDSA Cycle 1

Plan: Baseline audit findings and multidisciplinary discussions identified reduced patient mobility as a key concern. Barriers included lack of explicit mobility goals, reliance on physiotherapy-led mobilization, and the presence of invasive devices such as intravenous lines and urinary catheters.

Do: Two interventions were implemented: (i) Promotion of early mobilization during morning handovers and (ii) review and removal of unnecessary clinical devices that limited patient movement.

Study: Mobilization data were collected daily following implementation. Informal feedback was obtained from three nurses and three therapists regarding feasibility and staff engagement. Outcomes were compared with baseline audit findings.

Act: Although mobilization rates improved, variability in documentation across shifts was observed. A structured documentation tool was therefore developed for the next cycle.

PDSA Cycle 2

Plan: The second cycle aimed to enhance the consistency and sustainability of mobilization practices through structured documentation and increased staff awareness.

Do: Two additional interventions were introduced: (i) Implementation of a bedside “sit-out” chart to document daily mobilization. The charts were primarily maintained by nursing staff and were reviewed during routine ward rounds, multidisciplinary discussions and handovers when appropriate and (ii) display of educational posters highlighting the benefits of early mobilization.

Study: After one week, sit-out chart completion and mobilization rates were reviewed. Feedback was again obtained from three nurses and three therapists regarding usability and visibility of the interventions.

Act: Educational messages were reinforced during handovers, and the sit-out chart was streamlined to address initial completion issues. Plans were made for periodic re-audit to evaluate long-term sustainability.

Ethical considerations

This project was conducted as a clinical audit and service evaluation aimed at improving routine patient care. No identifiable patient data were collected and no interventions deviated from standard clinical practice. In accordance with local institutional policy, formal ethical approval and individual patient consent were not required.

## Results

Baseline audit findings

At baseline, the mean number of patients mobilizing out of bed by 11:00 a.m. was 7.73, representing 30% of eligible patients (median: 7). Most patients remained in bed during the morning period despite the absence of clear medical contraindications to mobilization.

Informal feedback from ward staff identified several factors contributing to low mobilization rates. These included the absence of explicit mobility goals during morning handovers, competing clinical priorities on the ward, and the presence of invasive devices such as intravenous lines and urinary catheters that limited patient movement.

PDSA Cycle 1 Outcomes

Following the implementation of Cycle 1 interventions, which emphasized early mobilization during morning handovers and encouraged the removal of unnecessary barriers to mobility, an improvement in early mobilization was observed.

The mean number of patients mobilizing out of bed increased to 14.71, corresponding to 57% of eligible patients (median: 14). Staff feedback indicated improved awareness of mobility as an important component of daily care and increased encouragement for patients to sit out of bed during the morning period. However, variability in documentation practices across different shifts was noted.

PDSA Cycle 2 Outcomes

During the second PDSA cycle, a sit-out chart and educational posters were introduced to improve documentation and reinforce awareness regarding early mobilization.

During this phase, the mean number of patients mobilizing out of bed was 13.14, corresponding to 51% of eligible patients (median: 14). Review of the sit-out charts demonstrated regular documentation of patient activity. Staff reported that the charts served as effective reminders to encourage mobilization, while educational posters increased awareness among staff, patients, and family members about the risks of hospital-associated deconditioning.

Hence, across the two PDSA cycles, early mobilization rates increased compared with baseline audit data. The proportion of patients mobilizing out of bed rose from 30% at baseline to 57% during Cycle 1, representing a 27% absolute improvement, and remained higher than baseline during Cycle 2 (51%) following implementation of structured documentation and educational prompts (Table 2).

**Table 2:** Early Mobilization Across Audit Phases PDSA: Plan-Do-Study-Act

Audit Phase	Mean Number Mobilizing by 11:00 a.m.	Percentage of Eligible Patients	Median
Baseline Audit	7.73	30%	7
PDSA Cycle 1	14.71	57%	14
PDSA Cycle 2	13.14	51%	14

No adverse events related to increased mobilization were reported during the audit period.

## Discussion

The two-cycle audit provided strong evidence that a targeted multi-disciplinary strategy can significantly improve early mobilization among elderly inpatient populations. We found that there was a significant increase in the number of patients being mobilized early into their hospital admission (in comparison to the baseline audit) after our intervention strategies were implemented. Specifically, there were increases noted in the number of patients sitting out of bed for meals and engaging in ambulatory practice within the first 48 hours of admission after the interventions were put into place. Each PDSA cycle built on the last, and by the second cycle, our mobilization rates had improved markedly relative to the original baseline. These positive trends suggest that the changes made were effective in altering how clinicians practiced on the unit on a day-to-day basis. It is demonstrated that prolonged bed rest in older adults contributes to deconditioning and loss of independence, highlighting the importance of promoting early mobilization during hospital admission [17]. In summary, the results of our audit suggest that our goal of making mobility a routine component of care was at least partially met and that by the end of the study, mobilizing patients early became the standard of care rather than an exception.

Interpretation and mechanisms

Improvements in mobilization on our ward can be attributed to several small, low-cost approaches that, together, changed both the attitudes of and the processes utilized by staff. One key factor contributing to this change was the formalized morning handover with a focus on patient mobility. By making mobility goals an explicit component of the daily discussion between the interdisciplinary team, all staff members were aware of a patient's "mobility plan" from the beginning of their day. This approach to structuring the daily conversation about mobility may have fostered a sense of accountability and priority among the staff. Nurses, healthcare assistants, and physicians now had clear directions regarding a patient's mobilization needs, and physicians appeared more inclined to consider mobilization as an important component of a patient's overall treatment plan (e.g., ordering earlier discontinuation of IV drips, reviewing a patient's analgesic regimen to allow for comfortable physiotherapy).

Another important change was the removal of any non-essential indwelling lines and catheters. Indwelling devices are generally recognized as deterrents to patient movement. A catheter can limit a patient's ability to leave their bed and may send a subtle message to the patient and the health care provider that the patient is too ill to safely walk to the toilet. Reducing the number of indwelling devices used on our unit not only reduced the number of physical barriers to patient movement but also conveyed to both patients and staff that ambulation was acceptable and anticipated when clinically appropriate. This portion of our study was in alignment with best practices in the care of older adults. Avoidance of bed-associated devices (when possible) is a widely accepted strategy to promote mobility and to prevent other problems associated with prolonged bed rest (e.g., catheter-acquired infection).

The introduction of sit-out charts provided a continuous feedback loop that reinforced the desired behavior. The charts made mobilization visible and measurable. Staff members could easily see whether a patient had been mobilized during the day and could intervene accordingly. Access to mobilization records enabled patients to monitor their progress and participate more actively in mobilization. In essence, the charts served as a constant reminder of the shared goal of keeping patients active. The educational posters and materials helped to create a supportive environment by increasing staff awareness of the rationale behind the changes that we made. The posters and materials referenced messages promoted by national initiatives, such as "End PJ Paralysis," the British Geriatrics Society's "Sit up, get dressed, keep moving" slogan, and helped to bring these messages to the local context of our ward [18,19].

Together, the various interventions developed for this study established multiple reinforcement points: verbal, handovers, physical (reduction of barriers to movement), presence of contextual posters and cues in the environment, which synergistically established a new standard of care for the interdisciplinary team on our unit. As a result, a culture shift occurred in how the interdisciplinary team cared for patients; mobilization became “everyone’s business,” rather than an afterthought or solely the realm of physiotherapists.

Our study supports the notion that even resource-neutral interventions can lead to meaningful improvements in care when they target fundamental care processes. This is consistent with other studies that relatively simple mobility programs can produce significant improvements. For example, Demirtas et al. reported that a surgical population that underwent targeted interventions (including staff education sessions and reminders via poster) experienced a significant increase in the day-of-surgery mobilization rates [20]. We similarly found that using coordinated, low-tech approaches to improve mobility resulted in breaking through the inertia of usual care in our elderly medical population. Notably, our findings are in line with the larger body of evidence related to improving mobility while in the hospital; maintaining mobility while in the hospital is associated with improved physical performance upon discharge, as well as reduced lengths of hospital stay and potentially improved long-term functional status [17]. Therefore, by establishing a greater rate of early mobilization, our study may contribute to those potential downstream benefits (although we did not collect data on outcomes like length of stay or post-discharge function). Importantly, we also found no increase in fall risk or other negative consequences of mobilizing patients early. This addresses one of the most cited concerns of staff members regarding changes to practice, that mobilizing patients early is unsafe and will increase the likelihood of future falls (and other complications such as pressure ulcers or deconditioning-related falls). Overall, the interventions in this study acted as catalysts for change, altering the standard of care in a manner that was sustainable and demonstrating that significant improvements in patient mobilization can occur without increased costs or personnel, simply by using resources more effectively within the existing team.

Cultural shift and multidisciplinary ownership

One of the most notable aspects of our project was the change in the ward's culture concerning mobilization. Prior to our project, we observed that health professionals often practiced role compartmentalization; for example, nurses may have waited until a physiotherapist arrived before mobilizing a patient, and physicians did not typically include mobility as an explicit consideration during rounds unless a patient was identified as being at high risk of falling or requiring rehabilitation. In contrast, by the end of our project, it was clear that a sense of shared responsibility among health professionals had developed. Nurses and healthcare assistants were more proactively encouraging patients to get out of bed early in the morning (and were often able to get patients into sitting positions in chairs prior to breakfast), and physicians were more frequently asking about mobility progress during rounds and establishing mobility goals as part of a patient's overall plan of care. Similarly, ancillary staff and family members visiting patients also appeared to be reinforcing the message (porters ensuring that patients had a chair to sit in while waiting for transport instead of using a stretcher, and bringing in appropriate footwear and day clothes for patients to wear). This aligns with the goal of national campaigns to encourage a shift in attitudes regarding prevention of deconditioning; each individual who interacts with a patient can play a role in promoting healthy behaviors to prevent deconditioning [21].

Our audit project provided a mechanism for creating this type of attitudinal change by normalizing the discussion of mobility at all levels of interaction. As such, cultural shifts related to this topic can be difficult to measure quantitatively; however, evidence supporting the sustainability of practice changes provides a tangible indicator that these types of cultural shifts exist. Specifically, our observation of ongoing "good habits" among the staff, including continued use of the sit-out charts and independent mobilization of patients on their own initiative during times between the formal audit cycles, indicates that the new norms have been adopted intrinsically by the team. Embedding the culture to support long-term success is critical. Geriatric society policy statements emphasize that preventing functional decline is not achieved through a single effort, but rather, through continuous and collective diligence. Consistent with this perspective, our experience suggests that once the entire team "bought in" to the value of mobilization, the positive outcomes that occurred were likely to continue beyond the duration of our audit project, and potentially even improve over time.

Relevance to literature and policy

The benefits from the QIs within our initiative are directly aligned with research on reducing hospital-associated deconditioning and geriatrics policies that exist today. The British Geriatrics Society, along with other related organizations, has identified deconditioning as an avoidable and significant risk associated with hospitalization. As a result of this, a variety of publications and campaigns have been created to help prevent this [21]. The "Sit up, get dressed, keep moving" campaign is an excellent example of the recommendations put forth by the society being implemented at the ward level through our project [18]. In essence, we were able to implement the spirit of the "Sit up, get dressed, keep moving" campaign at our ward level and demonstrate the viability of this approach; when you get patients to stand up and move, their functionality improves during hospitalizations. Furthermore, our goal of removing catheters and reducing bed rest is consistent with international best practices. The World Health Organization (WHO) has outlined the concept of healthy aging, largely in terms of the ability to maintain functional capacity, and mobility is a primary component of that [22]. By attempting to keep older adults mobile during episodes of acute illness, we are supporting the overall objectives of the concept of healthy aging and contributing to the creation of age-friendly hospital care. Additionally, hospital-associated deconditioning is currently recognized as a global public health issue in aging populations [15], and there is a call for health systems to ensure that older adults remain as active and independent as possible when hospitalized (such as part of the WHO Decade of Healthy Aging framework) [23]. Our study adds support to these calls by demonstrating that relatively simple changes can produce significant improvements in the day-to-day mobility of patients. Our study also reinforces prior evidence from systematic reviews and national guidelines recommending mobilization to decrease length of stay and post-hospital disability [17]. Overall, our QI project is grounded in the current paradigm of geriatric care and demonstrates locally derived evidence that supports the prevailing expert recommendations from groups including BGS, WHO, and others.

Challenges and lessons learned

Despite the successful implementation of this project, it was not without challenges, and the lessons learnt can be applied to future projects of this type. A major challenge was overcoming the status quo of established routine practice. Not everyone on the team was immediately receptive to taking on additional responsibilities to mobilize patients and/or to complete sit-out charts, especially in a high-workload environment. We attempted to overcome this by involving the team in the early stages of the project, explaining why we were doing this (utilizing the data collected from our baseline audit and patient stories to demonstrate the problem), and by providing immediate feedback on successes during the PDSA cycles. Leadership and role modeling were essential to this process. When senior nurses and the ward matron consistently demonstrated their commitment to this by leading by example (e.g., reminding staff about this during handover, assisting with transfers), it helped establish expectations and encouraged others to follow suit. An additional practical challenge was the variation in patient acuity and staffing levels. Days with multiple admissions and/or sick patients who required a higher level of care resulted in less time for mobility activities, despite our best intentions. We learned to be flexible and adapt the way we provided the intervention to meet the needs of the patients. This experience reinforced the notion that "early mobilization" is not a one-size-fits-all approach and should be tailored to the individual patient's abilities. For example, on a busy day, a highly dependent patient might be assisted to sit out in a chair for a shorter period, rather than attempting a long walk. Success should be conceptualized along a continuum, with even modest increases in activity considered beneficial. Staffing constraints remained a continuing concern, as transferring a dependent patient from the bed to a wheelchair typically requires assistance from at least two individuals. We learned to plan for mobility activities during handover to allow staff to coordinate to assist each other, and to utilize volunteer assistance when available, with proper guidance. However, we recognize that adequate staffing will continue to be a systemic issue beyond the scope of our project, and that long-term sustainability in mobilization will require sufficient numbers of personnel to aid when needed. We experienced minor issues with the sit-out chart usage, specifically initial variability in completion rates, i.e., some staff found them redundant. To enhance compliance, we simplified and streamlined to make it quicker to complete. The lesson learned here is that any new documentation or tool should clearly provide value to the end user and should be easy to use; otherwise, staff won’t adopt it among their numerous competing priorities.

Limitations

There are several important limitations of this study to acknowledge when evaluating our findings. First, due to its single-center, single-ward audit within an NHS hospital, the generalizability of our results to other wards or hospitals may be limited. For example, the successful implementation of our interventions was in large part because we had a predominantly frail elderly population with a high deconditioning risk; all of which was clearly apparent to our staff. Wards with shorter lengths of stay or younger populations may produce significantly different outcomes based on similar interventions. Second, we primarily assessed process-based measures (i.e., the percentage of patients who were mobilized by a certain time, the average number of days that patients spent out of bed, etc.) instead of assessing direct clinical outcomes. In addition, although reduction of unnecessary clinical tethers formed part of the intervention strategy, tether-specific quantitative data (e.g., prevalence of intravenous lines or urinary catheters before and after intervention) were not formally collected. Therefore, we did not assess muscle strength, gait speed, or functional independence at the time of discharge, nor did we collect information regarding post-discharge readmission rates or long-term functional status. While we believe that increased mobilization during inpatient care would likely result in improved clinical outcomes, as evidence suggests [17], we can provide no confirmation of these endpoints using our study. Additionally, we did not formally quantify the reduction in indwelling devices such as IV lines and catheters, though staff reported this was a major facilitator of improved mobility. Future research could assess functional ability or gather follow-up data after discharge to quantify the impact of QI projects on the individual level.

The closed-loop nature of this project resulted in a lack of a control group and implementation of an observational approach to tracking improvements over time. As a result, it is possible that other non-intervention-related factors or trends (such as a hospital-wide initiative aimed at preventing falls or a seasonal variation in the volume of patients admitted) could have independently affected mobilization rates, independent of our intervention periods. There are also a few other limitations to acknowledge. First, while we monitored the ward's incident reporting system and found no safety issues, we did not maintain a formal, independent log for falls or other adverse events. Second, we did not formally count the number of clinical tethers, such as IV lines and catheters, that were removed, even though this was a major part of our strategy. Finally, because our audit used a daily 'snapshot' at 11:00 a.m. to look at the whole ward, we did not track individual patient data like the average length of stay or the exact time from admission to first mobilization. Future studies could include these metrics to provide a more detailed look at the impact of such programs. Although we minimized confounding variables through limiting the duration and focus of our intervention periods and monitoring qualitative changes (none were significant), we cannot establish causality through this observational design like a randomized controlled trial design could. Nevertheless, the sequential increases in mobilization in response to our two intervention cycles and the temporal relationship between our specific interventions and these changes in practice support the inference that our actions were effective. Additionally, there may have been measurement bias as staff became more aware of mobility; the measured increase in mobilization may have been the result of better documentation of mobility. Nonetheless, we are confident that a real change in practice occurred beyond documentation alone, due to the magnitude of the improvement and the direct observation by the project leaders of more patients being mobile.

Implications and future directions

While there are many restrictions to our study, it demonstrates an idealized model of how a multidisciplinary, process-based QI project may improve the care provided to hospitalized older patients. It also illustrates that "standing up to deconditioning" is achievable on a busy NHS ward using teamwork and straightforward interventions that have been validated by clinical research. The sustainability of the cultural change in our ward suggests that the improvements can be maintained, especially if periodic reinforcement occurs (for example, continuing to discuss mobility in handovers and orienting new staff to the mobility culture). For all QI practitioners and for geriatric health care teams, our project also emphasizes the need to embed changes into existing processes and routines; as opposed to creating additional burdensome requirements, we simply added mobility to an existing requirement (the handover) and removed unnecessary requirements (catheterization) and replaced them with beneficial ones. Embedding the changes into existing processes and routines will likely be essential for sustaining the changes long-term. Additionally, this project may serve as a model that can be applied to other wards as well. Depending upon local needs, the exact strategies employed (use of sit-out charts, etc.) may vary from ward to ward; however, the fundamental strategy of engaging multiple disciplines and addressing modifiable barriers is widely applicable. Moreover, our project contributes to an increasing body of literature and momentum toward developing age-friendly hospitals. Nationally and internationally, there are large-scale initiatives underway to create elder-friendly hospital environments (such as the NHS's End PJ Paralysis Campaign [19], the Age-Friendly Health Systems Initiative [24]), and our project represents one example of such ideals being implemented at the ward level. Ultimately, our project illustrates that preventing loss of function is just as important as managing the medical condition that caused the patient to be hospitalized.

## Conclusions

Our findings support the notion that QI in geriatric care can effectively target processes of care to achieve better outcomes. This project focuses on process innovation in care delivery and organizational culture, rather than on the evaluation of new treatments or technologies. The appeal of implementing process innovations at a time when there are limited healthcare resources and high costs of innovation is that they are inexpensive and therefore practical. Simply changing how we collaborate and attend to basic needs such as mobility can significantly benefit the patient. In summary, the two-cycle audit on our geriatric care unit increased early mobilization through multidisciplinary collaboration and pragmatic interventions. This led to the creation of a sustainable shift in practice to one that is consistent with geriatric best practices, thereby positioning patients for optimal recovery. Through sustained commitment to these principles, hospital teams can continue to stand up to deconditioning, ensuring that older adults not only survive their hospital stay but also retain the functional ability to thrive afterward, which is the ultimate goal of age-attuned healthcare.
